# Evidence for quorum sensing and differential metabolite production by a marine bacterium in response to DMSP

**DOI:** 10.1038/ismej.2016.6

**Published:** 2016-02-16

**Authors:** Winifred M Johnson, Melissa C Kido Soule, Elizabeth B Kujawinski

**Affiliations:** 1 MIT-WHOI Joint Program in Oceanography/Applied Ocean Science and Engineering, Department of Marine Chemistry and Geochemistry, Woods Hole Oceanographic Institution, Woods Hole, MA, USA; 2 Department of Marine Chemistry and Geochemistry, Woods Hole Oceanographic Institution, Woods Hole, MA, USA

## Abstract

Microbes, the foundation of the marine foodweb, do not function in isolation, but rather rely on molecular level interactions among species to thrive. Although certain types of interactions between autotrophic and heterotrophic microorganisms have been well documented, the role of specific organic molecules in regulating inter-species relationships and supporting growth are only beginning to be understood. Here, we examine one such interaction by characterizing the metabolic response of a heterotrophic marine bacterium, *Ruegeria pomeroyi* DSS-3, to growth on dimethylsulfoniopropionate (DMSP), an abundant organosulfur metabolite produced by phytoplankton. When cultivated on DMSP, *R. pomeroyi* synthesized a quorum-sensing molecule, *N*-(3-oxotetradecanoyl)-l-homoserine lactone, at significantly higher levels than during growth on propionate. Concomitant with the production of a quorum-sensing molecule, we observed differential production of intra- and extracellular metabolites including glutamine, vitamin B_2_ and biosynthetic intermediates of cyclic amino acids. Our metabolomics data indicate that *R. pomeroyi* changes regulation of its biochemical pathways in a manner that is adaptive for a cooperative lifestyle in the presence of DMSP, in anticipation of phytoplankton-derived nutrients and higher microbial density. This behavior is likely to occur on sinking marine particles, indicating that this response may impact the fate of organic matter.

## Introduction

The carbon cycle is essential to the regulation of conditions on Earth from the climate to the pH buffering capacity of the ocean. Atmospheric carbon stores (750 Pg) are dwarfed by the 31 000 Pg of dissolved carbon present in the ocean, of which 660 Pg comprise dissolved organic matter. This complex pool of organic molecules contains substrates that serve as the primary catabolic energy source for heterotrophic bacteria, as well as compounds that are transferred among microbes to promote growth or cooperative community function. Because microbes have varying requirements for organic substrates, organic matter composition influences the microbial community and vice versa (Baña *et al.*, 2014; Sharma *et al.*, 2014). Some microbes are metabolically specialized, whereas other microbes are generalists that can adapt to a variety of conditions (Lauro *et al.*, 2009). Recent studies have documented complex relationships between primary producers and heterotrophic bacteria (for example, Amin *et al.*, 2015; Durham *et al.*, 2015). Experiments with the diatom *Thalassiosira pseudonana,* showed increased intracellular production of amino acids in response to co-culturing with the heterotroph *Dinoroseobacter shibae* (Paul *et al.*, 2012). In another study, the Roseobacter *Phaeobacter gallaeciensis* was found to produce phytotoxins in response to *p*-coumaric acid, an algal cell wall breakdown product (Seyedsayamdost *et al.*, 2011). More generally, vitamins such as cobalamin, biotin and thiamin are produced by some heterotrophic bacteria and assimilated by eukaryotic organisms that are auxotrophic for these essential biochemicals (Croft *et al.*, 2006). Likewise, organosulfur metabolites are required by some marine bacteria, such as the ubiquitous SAR11, which cannot reduce inorganic sulfate (Tripp *et al.*, 2008). Finally, some molecules, including algal break-down products and acyl homoserine lactones, act as infochemicals to signal events such as a nutrient pulse or population changes (Sandy and Butler, 2009; Seyedsayamdost *et al.*, 2011). Although these interactions between microbes and molecules occur on very small spatial scales, they culminate in the production and remineralization of 50–100 Pg of carbon annually (Martin *et al.*, 1987).

DMSP is a widely recognized intermediate in these types of interactions. It is produced predominantly by dinoflagellates, coccolithophores, diatoms and higher plants like the marsh grass *Spartina* (Stefels, 2000). Intracellularly, it is present at high concentrations ranging from 0.1 to 1 m (Stefels, 2000) and bulk particulate concentrations can reach 120 nm (Kiene and Slezak, 2006), making it a quantitatively significant organic substrate in the ocean (Simó *et al.*, 2009). Not surprisingly, it has been demonstrated to have a vital role in the marine food web as a source of reduced carbon and sulfur (González *et al.*, 2003; Simó *et al.*, 2009) and may act as an antioxidant in some organisms (Sunda *et al.*, 2002).

In the open ocean, dissolved DMSP concentrations are generally found in the sub-nanomolar range but can reach low nanomolar levels around a phytoplankton bloom and are likely elevated in the immediate wake of sinking aggregates of dying phytoplankton cells and/or fecal pellets (Yoch, 2002; Kiene and Slezak, 2006; Levine *et al.*, 2012). Consequently, DMSP is associated with the release of labile organic matter (Biddanda and Benner, 1997; Grossart, 2010; Stocker, 2012). Thus, heterotrophic organisms seeking hotspots of organic nutrients could reasonably use DMSP as a proxy for other, more complex, molecules. Indeed, previous work has shown that increased colonization occurs on DMSP-spiked agar particles relative to non-amended agar particles (Kiørboe *et al.*, 2002). Along these lines, DMSP acts as a chemo-attractant for organisms ranging from heterotrophic bacteria to herbivorous dinoflagellates (Seymour *et al.*, 2010). In one study, the presence of DMSP triggered the bacterial production of a plant growth hormone to promote growth of a phytoplankton species and thus augment organic matter production (Seyedsayamdost *et al.*, 2011).

Bacteria with relatively large genomes can take advantage of transient nutrient pulses and thus could be well-suited to respond to DMSP-derived signals. To examine the metabolic response of a heterotrophic bacterium to DMSP, we focus here on the metabolically flexible bacterium, *Ruegeria pomeroyi* DSS-3, a coastal isolate of the Roseobacter clade of marine α-proteobacteria (Moran *et al.*, 2004). *R. pomeroyi* possesses genes for a variety of metabolic strategies including lithoheterotrophy using carbon monoxide or sulfide, flagellar motility and transporters for algal osmolytes and carboxylic acids (Moran *et al.*, 2004). These attributes, which have been confirmed in culture, enable *R. pomeroyi* to exploit rapid chemical changes in its environment. In particular, it can catabolize DMSP via two different pathways and incorporate atoms derived from DMSP into its biomass (González *et al.*, 2003; Reisch *et al.*, 2013). There are also indications that DMSP may provide a signal to prime *R. pomeroyi*'s metabolism to utilize algal lysates and exudates through increased transcription of transporter genes as well as a potential quorum-sensing response (Bürgmann *et al.*, 2007). Thus, *R. pomeroyi* is likely to have the metabolic capability to respond to DMSP as an indicator of a shift in nutrient availability.

We examined *R. pomeroyi*'s relationship with DMSP by comparing its metabolic response to DMSP as a carbon source relative to propionate, a three-carbon carboxylic acid that lacks the dimethylsulfide functional group. If DMSP serves only as an organic substrate for *R. pomeroyi*, then the primary metabolic difference in our experiment should be a response to the reduced sulfur in DMSP (Reisch *et al.*, 2013) because propionate catabolism shares the last two steps of one of the DMSP catabolic pathways. Alternatively, if DMSP indeed functions as a signal to *R. pomeroyi* of changing environmental conditions, we would anticipate a suite of metabolic changes that facilitate adaptation to a DMSP-containing microzone.

The microzones in the ocean where DMSP is prevalent have several characteristics in common. Specifically, these microzones have higher nutrient availability compared with the surrounding water column, particularly organic substrates, which can provide nitrogen and phosphorus, and they host a dense microbial community whose members compete for, and exchange, valuable substrates and nutrients (Biddanda and Benner, 1997; Grossart, 2010). In response to these characteristics, *R. pomeroyi* and similar metabolically flexible bacteria could change their metabolism to take advantage of the shift in chemical regime. Here we examine three hypotheses using intracellular and extracellular metabolic profiles. First, higher cell densities facilitate cell–cell communication (or quorum sensing) and resultant coordinated behaviors, and thus we expect to resolve and identify chemical signaling molecules. Second, the most energetically favorable means of obtaining nutrients, particularly nitrogen, would be to acquire them directly from the labile organic matter ubiquitous in the microzones (Kirchman *et al.*, 1989, 2003; Vallino *et al.*, 1996). Bürgmann *et al.* (2007) demonstrated that *R. pomeroyi* increases transcription of genes associated with transporters for nitrogen-containing organic substrates when grown on DMSP. We anticipate that a change in nutrient acquisition strategy would also result in differences in the concentrations of regulatory metabolites. Last, we suggest that a mutually beneficial exchange of high-value metabolites with other bacteria or phytoplankton would occur (Amin *et al.*, 2015; Durham *et al.*, 2015), leading to an adaptive and cooperative lifestyle similar to that observed during bacterial colonization of organic particles (Grossart *et al.*, 2003).

Our study examines the impact of DMSP relative to propionate on metabolite production and organic matter excretion by *R. pomeroyi*. We used complementary untargeted and targeted metabolomics methods to analyze the suite of molecules produced by *R. pomeroyi*, both within the cells and in the external medium. These metabolites ranged from those associated with primary metabolism, which shed light on fundamental cellular processes, to those associated with secondary metabolism, which help us understand the cell's response to shifting environmental conditions.

## Materials and methods

The complete methods are given in the Supplementary Information. Briefly, *R. pomeroyi* DSS-3 [DSM 15171] was grown in the dark at 23 °C in 0.2 μm-filtered (Omnipore (PTFE), EMD Millipore, Billerica, MA, USA) seawater amended with 3 mm carbon (1 mm sodium propionate or 0.6 mm DMSP), 4 mm ammonium chloride, 30 nm monosodium phosphate, 100 nm ferrous chloride-ethylenediaminetetraacetic acid, 100 nm zinc chloride, 100 nm manganese(II) chloride, 1 nm cobalt(II) chloride and 1 ml l^−1^ medium of f/2 vitamin solution. Duplicate cultures were killed at 0, 32, 38(propionate)/43(DMSP), 48, 60 and 72 h with cell counts in the range of 10^6^–10^7^ cells ml^−1^ (Supplementary Figure S1a). The third time point was offset between the propionate and DMSP treatments so that the same cell abundance was sampled in each treatment. Cells were captured on a 0.2 μm filter (Omnipore (PTFE), EMD Millipore) using a combusted glass vacuum filtration apparatus (vacuum never greater than 10 mm Hg). Filters were frozen in cryovials at −80 °C immediately after filtration. Intracellular metabolites were extracted from filters using cold 40:40:20 acetonitrile:methanol:water+0.1 m formic acid solution, adapted from previously published protocols (Rabinowitz and Kimball, 2007). Half of the extract was further extracted using Agilent Bond Elut PPL (styrene-divinylbenzene polymer) solid-phase extraction resin for untargeted analysis. The extracellular metabolites were extracted from acidified filtrate (pH 2–3) using PPL cartridges (Dittmar *et al.*, 2008; after modification by Longnecker, 2015).

All the samples were separated with the same liquid chromatography method on a C18 reversed-phase column with a water and acetonitrile gradient (Kido Soule *et al.*, 2015). The targeted method used a Thermo Scientific triple quadrupole mass spectrometer with a heated electrospray ionization source. Untargeted analyses were conducted using a hybrid linear ion trap—7T Fourier transform ion cyclotron resonance mass spectrometer (FT-ICR-MS; LTQ FT Ultra, Thermo Scientific) with an electrospray ionization source.

The targeted data were processed using Xcalibur (Thermo Scientific software) and a 5 to 7-point standard curve for relative quantification. The untargeted mass analysis files were converted from Thermo RAW files to mzML files using MSConvert (Chambers *et al.*, 2012). The resulting data were processed using XCMS (Smith *et al.*, 2006; Tautenhahn *et al.*, 2008; Benton *et al.*, 2010) and CAMERA (Kuhl *et al.*, 2012). Peak picking and alignment were carried out separately for the intracellular and extracellular samples generating a list of features (defined as a unique combination of mass-to-charge value (*m/z*) and retention time) for each sample set (Longnecker *et al.*, 2015). Feature lists were refined through quality control requirements and are reported as mass spectral peak area normalized to cell abundance (intra- and extracellular; peak area per cell) or volume (extracellular; peak area per liter). Features in the untargeted data were putatively identified by comparison of *m/z* to the METLIN database (Smith *et al.*, 2005), and if available, by comparison of the associated fragmentation spectrum with experimentally generated fragmentation patterns in the METLIN database or to *in silico*-derived fragmentation patterns from MetFrag (Wolf *et al.*, 2010). When possible, commercially available analogs for putatively identified metabolites were analyzed using identical protocols.

Differences in the concentration of individual metabolites between the substrate treatments were compared with analysis of variance. In the intracellular targeted data, *P*-values <0.0013 were considered significant due to the Bonferroni correction for multiple comparisons.

Total organic carbon (TOC; unfiltered) and dissolved organic carbon (DOC; 0.2-μm filtered) samples were acidified and then analyzed using a Shimadzu TOC-V_CSH_ Total Organic Carbon Analyzer, according to standard practices. Methanethiol and dimethylsulfide were measured using gas chromatography (adapted from Levine *et al.* (2012)).

## Results

### *R. pomeroyi* growth parameters

*R. pomeroyi* cells exhibited similar growth rates (0.1 h^−1^) on both substrates (DMSP or propionate), and remineralization of the organic carbon substrate was confirmed by the diminishing concentrations of DOC and TOC in the media (Supplementary Figure S1b). Methanethiol and dimethylsulfide (DMS), which are products of two different DMSP degradation pathways, were produced during growth on DMSP (Supplementary Figure S1c; Reisch *et al.*, 2011, 2013). In this study, we did not quantify the two gases as we only sought confirmation that both pathways were active.

### Quantifying the metabolome (targeted metabolites)

**Figure 1:**
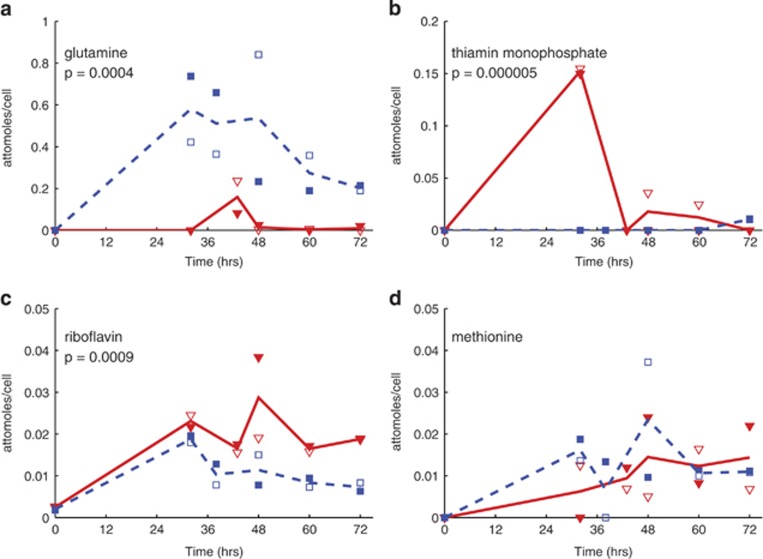
Intracellular metabolite profiles indicate differential concentrations of glutamine and two B vitamins while methionine concentrations are the same. All metabolites presented here were measured in the targeted method: (**a**) glutamine, (**b**) thiamin monophosphate, (**c**) riboflavin, (**d**) methionine. Each line reflects the average of two biological replicates, shown in open and closed symbols: red triangles—DMSP treatment; blue squares—propionate treatment. The *P*-value from an analysis of variance test is reported for metabolites with a significant difference in concentration between the substrate treatments across all time points.

**Figure 2:**
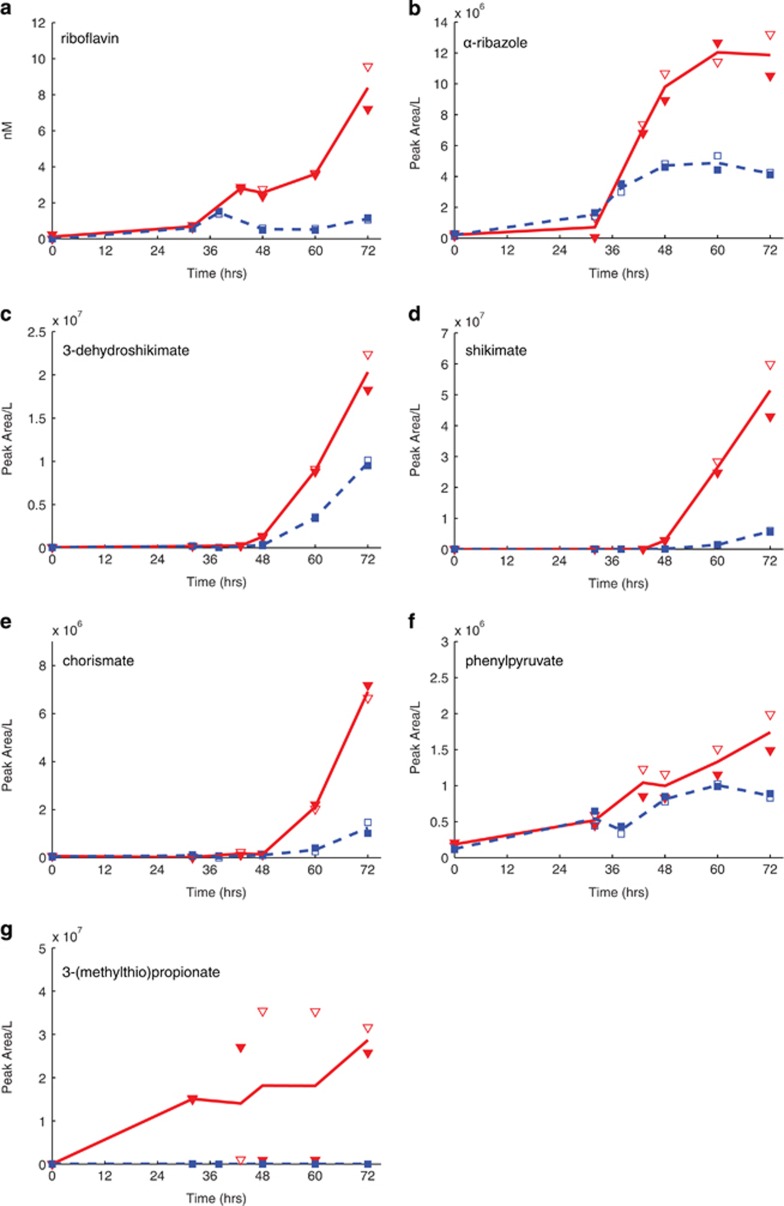
Energetically high-value metabolites are found in the medium at higher concentrations in the DMSP treatment, along with a metabolite associated with DMSP degradation. Each subplot shows the extracellular concentration profile of a metabolite over time. In each subplot, the line reflects the average of two biological replicates, shown in open and closed symbols: red triangles—DMSP treatment; blue squares—propionate treatment. (**a**) Riboflavin, measured in the targeted method and reported in nm. The remaining metabolites were measured in the untargeted method and the concentration is reported as peak area per liter. (**b**) α-Ribazole, (**c**) 3-dehydroshikimate, (**d**) shikimate, (**e**) chorismate*, (**f**) phenylpyruvate*, (**g**) 3-(methylthio)propionate* (an asterisk (*) denotes putative metabolite identifications with an identification ranking of 3 (see Supplementary Table S3).).

In targeted metabolomics, the goal is to obtain relative concentrations of a pre-defined list of organic compounds. Most of the intracellular and extracellular metabolites quantified by our method showed no significant difference between the substrate treatments. For example, intracellular methionine concentrations were similar in both treatments (Figure 1d). The measured compounds in the targeted method include vitamins, amino acids, nucleotides and precursors to these molecules, implying that primary cellular processes remained similar regardless of substrate (see Supplementary Figure S2 for the list of intracellular metabolites quantified). However, within the targeted data encompassing both intra and extracellular samples, significant changes were observed for one of the amino acids (glutamine), several vitamins and intermediates in cellular nitrogen and sulfur cycling. Intracellular glutamine concentrations by time point ranged from 3.5 to 157 times lower during growth on DMSP relative to growth on propionate (Figure 1a). This varied by time in the growth curve and the concentrations were particularly low in the DMSP treatment. Conversely, the B vitamins thiamin monophosphate (phosphorylated vitamin B_1_) and riboflavin (vitamin B_2_) were both elevated within the cell at some time points in the DMSP treatment (Figures 1b and c, respectively). Extracellularly, the maximum concentration of riboflavin was almost eight times higher in the DMSP treatment (Figure 2a).

**Figure 3:**
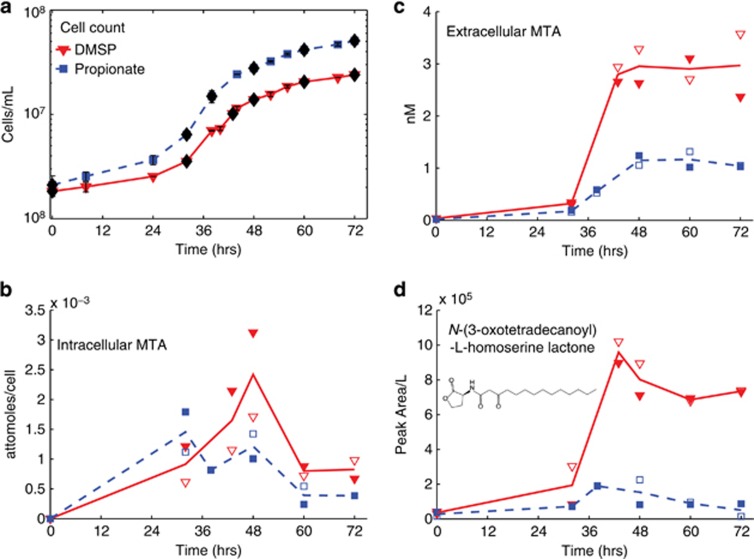
MTA linked to AHL production. (**a**) Growth curve for *R. pomeroyi*. Red solid line is the DMSP treatment and the blue dashed line is the propionate treatment. Error bars represent one standard deviation of replicate bottles. In the remaining subplots, the line reflects the average of two biological replicates, shown in open and closed symbols: red triangles—DMSP treatment; blue squares—propionate treatment. (**b**) The intracellular concentrations of MTA over time, normalized to the number of cells. (**c**) The extracellular MTA concentrations over time. (**d**) Extracellular concentrations of the quorum-sensing compound *N*-(3-oxotetradecanoyl)-l-homoserine lactone. The structure of *N*-(3-oxotetradecanoyl)-l-homoserine lactone is shown in the inset of **d**.

The intracellular concentrations of 5′-deoxy-5′-methylthioadenosine (MTA), a sulfur-containing metabolite, were not significantly different between the two treatments (Figure 3b). However, in the extracellular fraction, MTA concentrations were approximately three times higher in the DMSP treatment than in the propionate treatment (Figure 3c), indicating increased production and release of MTA into the medium during DMSP-based growth. Given its sulfur content, the elevated MTA concentration could be derived from excess sulfur generated during DMSP catabolism. We tested this hypothesis with an experiment that monitored MTA concentrations in three treatments: 100% propionate, 90% propionate/10% DMSP and 100% DMSP, where all the treatments contained the same amount of dissolved organic carbon (3 mm). When normalized to cell abundance, the extracellular MTA concentrations in the full DMSP and the 10% DMSP treatments were seven and six times higher, respectively, than in the propionate treatment (Supplementary Figure S3a). Therefore, despite the 90% reduction in sulfur, there was not a corresponding decrease in cell-normalized MTA concentration.

### Profiling the metabolome (untargeted metabolites)

The goal of the untargeted method is to profile as much of the metabolome as possible. Unlike the targeted method it is only semi-quantitative, and relative abundances cannot be converted into molar quantities. However, metabolites measured in both the targeted and untargeted methods show highly similar instrument responses between the two methods. This is shown here for MTA (Supplementary Figure S3b) and previous work has demonstrated this for other metabolites (Fiore *et al.*, 2015). This confirms that relative abundances of features (that is, molecules defined by a unique combination of *m/z* value and retention time) within the untargeted data set can be compared and statistically evaluated.

Hundreds of intracellular features were resolved with the untargeted metabolomics method (Supplementary Table S1). Most of these (43%, negative ion mode; 96%, positive ion mode) were detected in both substrate treatments but their relative abundances illustrate more striking differences between the treatments (Supplementary Table S2). At 60 h, a majority (86%, negative ion mode; 64%, positive ion mode) of the intracellular features were five times higher in the propionate treatment. In contrast, a minority (9%, negative ion mode; 17%, positive ion mode) of the intracellular features were at least five times higher in the DMSP treatment relative to the propionate treatment (Supplementary Table S2).

Hundreds of extracellular features were similarly observed in these samples (Supplementary Table S1). A non-metric multidimensional scaling analysis of the extracellular metabolic profiles shows that samples cluster more by cell density and/or growth phase than by substrate treatment (Supplementary Figure S4). Carbon substrate is of secondary importance but has a discernible impact, particularly at later time points. A comparison of peak area per cell of the individual extracellular features at 60 h shows that, depending on substrate and ionization mode, between 19 and 36% of these features were at least five times higher in one of the substrate treatments (Supplementary Table S2).

### Identification of features in the untargeted data set

The structural diversity of metabolites and the gaps in the characterization of metabolic pathways present major challenges to the identification of features in the untargeted data set. Our approach was to identify as many features as possible within the ranking system proposed by Sumner *et al.* (2007) and described in Longnecker *et al.* (2015). Metabolites are identified with increasing confidence first by an *m/z* match, then a fragmentation match to a database, and, for the highest confidence level, comparison to an authentic standard analyzed with our method to confirm *m/z* value, retention time and fragmentation pattern. In some cases, putative identifications of metabolites with only an *m/z* match can be considered more significant if intermediates in the same metabolic pathway are identified at higher rankings (Suhre and Schmitt-Kopplin, 2008). These stringent requirements result in a relatively small subset of metabolites that we can reliably identify. However, we can expect to be able to identify a larger set of metabolites in future studies as the field of metabolomics matures; these additional identifications will add greater nuance to our understanding of metabolic responses and their ecological implications.

**Figure 4:**
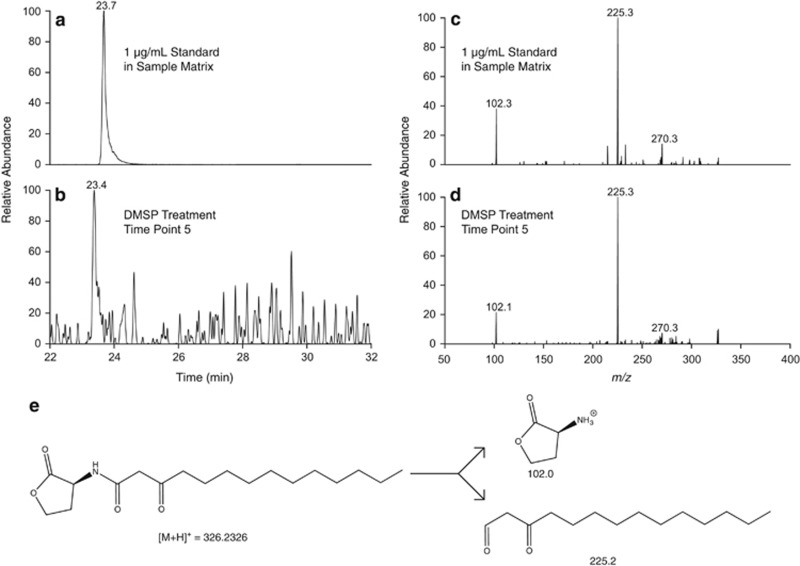
AHL identification. *N*-(3-Oxotetradecanoyl)-l-homoserine lactone commercial standard was spiked into original extracellular extract from the experiment approximately 6 months after the experiment. (**a**) Extracted ion chromatograms displaying the mass range within 1 Dalton of the mass of *N*-(3-oxotetradecanoyl)-l-homoserine lactone. (**b**) The same data in the original experimental sample. The difference in retention time was confirmed to be owing to changes in the column over the 6-month lag between analyses. *N*-(3-Oxotetradecanoyl)-l-homoserine lactone fragments into a lactone head group (102.0 *m/z*) and a tail group fragment (225.2 *m/z*). (**c**) Fragmentation of the commercial standard. (**d**) Fragmentation of the feature identified in the experiment. (**e**) The structure of *N*-(3-oxotetradecanoyl)-l-homoserine lactone and its primary fragments.

We focused our most rigorous identification efforts, including purchasing standards, on features that were differentially produced in the two treatments. MTA was quantified by our targeted method and found to be elevated in the extracellular fraction of the DMSP growth treatment; it was similarly detected in the untargeted method (Supplementary Figure S3b). MTA can be produced by two different pathways, as a by-product of spermidine/spermine synthesis or acyl homoserine lactone (AHL) biosynthesis (Parveen and Cornell, 2011; Supplementary Figure S5). Structurally, AHLs consist of a lactone head-group linked by an amide to a carbon chain of varying lengths and degrees of saturation (Figure 3d, inset). AHLs are a class of quorum-sensing chemicals and *R. pomeroyi* is known to produce three different AHL compounds (Moran *et al.*, 2004). We searched the untargeted data set for predicted *m/z* values for all AHLs up to a 16-carbon chain length. We identified *N*-(3-oxotetradecanoyl)-l-homoserine lactone in our data set, based on exact mass and fragmentation pattern matches in the METLIN database (Smith *et al.*, 2005). Its size was consistent with an AHL observed by Moran *et al.* (2004). Comparison with an authentic standard revealed a positive identification for this compound (Figure 4). The identified AHL is elevated in the extracellular medium by a factor of 8–15 in the DMSP treatment at the last four time points of the experiment (Figure 3d), where MTA concentrations are also high.

We also putatively identified precursors to the cyclic amino acids (tryptophan, phenylalanine and tyrosine) including chorismate, shikimate, phenylpyruvate and their associated intermediates (Supplementary Table S3; Supplementary Figure S6). These features occur at higher relative concentrations in the DMSP treatment (Figure 2c–f). Other putative identifications include the first metabolite in the DMSP demethylation pathway, 3-(methylthio)propionate, which was exclusively observed in the DMSP treatment in both extracellular and intracellular fractions (Figure 2g; Supplementary Table S3 and S4). In addition, in the untargeted data, α-ribazole, a component of vitamin B_12_, was putatively identified and found to accumulate in the media over time (Figure 2b; Supplementary Table S3). While the current annotations of *R. pomeroyi*'s genome do not predict direct synthesis of α-ribazole, its production may result from an un-annotated biosynthetic pathway or non-enzymatic degradation of vitamin B_12_.

## Discussion

The primary goal of this study was to examine the difference between the two growth substrates (that is, DMSP and propionate) beyond the obvious differences in direct degradation of the carbon substrate. We hypothesized that these differences would be observed either in sulfur-containing metabolites (Thiel *et al.*, 2010) or in metabolites related to a re-tooling of metabolism for a more cooperative lifestyle (Geng *et al.*, 2008; Seyedsayamdost *et al.*, 2011). While a few sulfur-containing metabolites, such as MTA, had a higher abundance in the DMSP treatment, we did not find any indication of far-reaching effects on sulfur-containing metabolites under DMSP-growth. For instance, an essential metabolite such as methionine, a sulfur-containing amino acid, had equivalent concentrations in the two treatments (Figure 1d) suggesting its intracellular concentration is highly regulated and insensitive to the influx of sulfur. Thus, rather than an impact on only S-containing molecules, we observed a broad shift in metabolite patterns, suggesting a pervasive impact of DMSP on the *R. pomeroyi* metabolome.

### DMSP degradation

The fate of DMSP during degradation by *R. pomeroyi* has been extensively studied (Reisch *et al.*, 2013) and the concentrations of metabolites within the DMSP degradation pathways are consistent with previous genomics research. On the basis of the metabolites measured, both the DMSP demethylation and the cleavage pathways were active in the DMSP treatment (Figure 2g; Supplementary Table S4; Supplementary Figure S1c). After the production of these molecules, the atoms derived from DMSP are incorporated into intermediates within central carbon metabolism and are impossible to discern relative to propionate metabolism (without isotope labeling).

### MTA as a proxy for possible quorum sensing in *R. pomeroyi*

MTA, which accumulates in the DMSP treatment, is an intermediate in the methionine salvage pathway, which allows the cell to recycle sulfur atoms for methionine synthesis. *R. pomeroyi* lacks the genes that complete the classic methionine salvage pathway, suggesting that excess MTA produced by *R. pomeroyi* would be excreted rather than recycled. MTA is a by-product of AHL synthesis and of spermidine/spermine synthesis (Supplementary Figure S5; Parveen and Cornell, 2011). *R. pomeroyi*'s genome suggests that it can synthesize spermidine and spermine, but that this occurs through an alternative pathway that does not produce MTA. Therefore, of the characterized pathways that generate MTA as a by-product, the *R. pomeroyi* genome is only known to contain genes required for AHL synthesis (Supplementary Figure S5). Furthermore, Bürgmann *et al.* (2007) have demonstrated increased transcription of a gene annotated as a ‘transcriptional regulator, LuxR family' (*luxR* is the AHL receptor gene) in response to DMSP. Although *luxR* and *luxI* (the AHL synthase gene) did not show increased transcription in the Bürgmann *et al.* (2007) study, the regulation of these systems is complex, as shown in species closely related to *R. pomeroyi* (Zan *et al.*, 2014), and might not be visible in the transcriptome for a variety of reasons. On the basis of this genomic information, MTA was most likely found at higher concentrations in the DMSP treatment owing to increased AHL synthesis. This hypothesis was supported in the untargeted data by the detection of one AHL, *N*-(3-oxotetradecanoyl)-l-homoserine lactone. The coherence of MTA and AHL dynamics suggests that the elevated MTA concentrations are linked to increased synthesis of *N*-(3-oxotetradecanoyl)-l-homoserine lactone and thus the presence of a possible quorum-sensing response by *R. pomeroyi* to elevated DMSP concentrations. A linear decrease in MTA concentration was not observed between a culture grown on 100% DMSP and one grown on 10% DMSP/90% propionate, further implying that the elevated accumulation of MTA in the DMSP treatment is not simply owing to the increased reduced sulfur pool, but instead is the result of a regulatory response triggered by DMSP.

Phenotypes regulated by a quorum-sensing system generally depend on a high density of cells to be effective. Quorum sensing can regulate activities such as antibiotic production, exoenzyme synthesis or biofilm formation (Miller and Bassler, 2001). *R. pomeroyi* can produce exoenzymes to break down large biopolymers (Christie-Oleza *et al.*, 2015) and has genes for flagellar motility, which can be regulated by a quorum-sensing system (Moran *et al.*, 2004). The microzones with likely elevated DMSP concentrations generally have high microbial population densities. With a population-dependent cell–cell communication pathway, such as AHL-based quorum sensing, *R. pomeroyi* could coordinate a shift in its metabolism to exploit the increase in organic nutrient availability. This hypothesis is consistent with previous characterization of a quorum-sensing phenotype of *Ruegeria* sp. KLH11, a marine sponge symbiont (Zan *et al.*, 2012), and with recent findings of measurable quantities of a number of AHLs in association with sinking marine particles (Hmelo *et al.*, 2011). Further work will be needed to understand the specificity of *R. pomeroyi*'s response to DMSP including experiments that evaluate *R. pomeroyi* metabolic (and transcriptomic/proteomic) profiles under exposure to the AHL identified here, as well as experiments with *luxR/luxI* knock-out mutants to isolate the affected metabolic pathways.

### Glutamine as a proxy for altered nitrogen assimilation strategy

It is particularly striking that intracellular glutamine concentrations were relatively reduced in the DMSP treatment because it is a metabolite that is highly sensitive to nitrogen availability and is integral to a cell's nitrogen cycle (Brauer *et al.*, 2006). Glutamine can serve as a nitrogen storage compound because it is produced when ammonium is assimilated into the cell. Once present, glutamine is an essential precursor for the synthesis of other amino acids and donates an amino group during the synthesis of purines, pyrimidines, amino sugars and NAD^+^ (Reitzer, 2003). High glutamine concentrations indicate that sufficient ammonium concentrations are present. In contrast, low glutamine concentrations signal a decline in ammonium concentrations and result in the upregulation of alternative nitrogen assimilation pathways. This has been observed not only in *E. coli*, but also in organisms such as *Saccharomyces cerevisiae* (Brauer *et al.*, 2006). In our experiments, excess ammonium was present in the growth media in both treatments, and thus inorganic nitrogen availability can be discounted as a controlling factor of intracellular glutamine concentrations.

Through a cascade of signals, glutamine also regulates alternative forms of nitrogen acquisition. In *E. coli,* for example, low glutamine concentrations are correlated with increased transport of organic nitrogen substrates such as amino acids, peptides and polyamines into the cell to offset the decline in extracellular ammonium (Zimmer *et al.*, 2000; Chubukov *et al.*, 2014). While the regulation of nitrogen assimilation has not been studied in *R. pomeroyi*, *R. pomeroyi's* genome annotations indicate the presence of genes for both pathways for ammonium assimilation (glutamate dehydrogenase and glutamine synthetase) as well as for the nitrogen regulatory protein P-II and a uridylyltransferase, all of which would be required to regulate nitrogen assimilation in a manner similar to *E. coli*. Bürgmann *et al.* (2007) previously observed that *R. pomeroyi*'s transcriptional response to DMSP involved upregulation of genes specifically associated with amino acid and polyamine transport. This accumulated evidence suggests that *R. pomeroyi* altered its nitrogen assimilation strategy in response to the presence of DMSP. Specifically, we hypothesize that *R. pomeroyi* is regulating intracellular glutamine concentrations to promote organic nitrogen uptake and utilization. Scavenging nitrogen from organic sources rather than utilizing ammonium could benefit *R. pomeroyi* by decreasing the cellular energy expenditure associated with ammonium assimilation and/or full biosynthesis of nitrogenous metabolites. To test this further, experiments are needed that compare the uptake of organic nitrogen sources and the differential transcription of nitrogen assimilation genes, in both the presence and absence of DMSP.

### Production of extracellular metabolites with potential for microbial cross-feeding

Reduced glutamine concentrations can also affect the diverse biosynthetic pathways that rely on this metabolite. For example, during nitrogen starvation in *E. coli*, decreased glutamine concentrations result in lower tryptophan concentrations and accumulation of phenylpyruvate, a precursor of phenylalanine, owing to decreased *de novo* synthesis of phenylalanine from phenylpyruvate (Brauer *et al.*, 2006). Tryptophan shares a common precursor, chorismate, with the other cyclic metabolites phenylalanine, tyrosine, 2,3-dihydroxybenzoic acid and 4-aminobenzoic acid (Supplementary Figure S6). In our experiment, intracellular and extracellular tryptophan concentrations remain the same in both treatments, while its precursors, 3-dehydroshikimate, shikimate, chorismate and phenylpyruvate, all accumulate in the extracellular fraction of the DMSP treatment. This suggests that the precursors for tryptophan synthesis were not being utilized by *R. pomeroyi* either owing to decreased synthesis of the amino acid or to increased production of the precursors.

The cellular release of these metabolically high-value biosynthetic intermediates supports the third component of our hypothesis, namely that switching to a cooperative lifestyle triggered by DMSP would require the release of some metabolites that could be used in cross-feeding. The increased extracellular concentrations of precursors like chorismate and phenylpyruvate could provide labile intermediates to surrounding microbes with incomplete amino acid biosynthetic pathways; these organisms might in turn produce valuable metabolites that *R. pomeroyi* could use. According to the annotated *R. pomeroyi* genome, *R. pomeroyi* can synthesize all amino acids except asparagine and possibly histidine. The analysis of 3062 available bacterial genomes in the Integrated Microbial Genomes database reveals that biosynthetic capability in bacteria ranges from 0 to 20 amino acids, averaging only 7.9 complete amino acid biosynthetic pathways. Bacteria are most commonly auxotrophic for tyrosine, phenylalanine, lysine or histidine. In 124 eukaryote genomes, complete biosynthetic pathways are present, on average, for only 4.1 out of 20 amino acids with auxotrophy most commonly found for histidine, lysine, serine or leucine and no species able to synthesize more than 14 of the 20 essential amino acids (Mee and Wang, 2012). The enhanced excretion of these biosynthetically high-value cyclic intermediates under DMSP-derived growth may be linked in part to changes in nitrogen assimilation strategy used by *R. pomeroyi*. However, these metabolites would also serve as valuable currency for microbial cross-feeding as evidenced by the ubiquity of incomplete *de novo* amino acid synthesis pathways.

Other metabolites that could be used in microbial cross-feeding are riboflavin (vitamin B_2_) and α-ribazole, a component of vitamin B_12_. Both of these molecules accumulated in the extracellular matrix of the DMSP treatment (riboflavin to 8X and α-ribazole to 3X at 72 h; Figure 2a and b). It is reasonable to speculate that the availability of a precursor such as α-ribazole, as well as the vitamin, riboflavin, would be valuable within a dense microbial consortium. For example, auxotrophy for α-ribazole has been demonstrated in the bacterium, *Listeria innocua*, which has a specific transporter to acquire this compound from its environment (Gray and Escalante-Semerena, 2010). Although α-ribazole, as well as the cyclic amino acid precursors mentioned earlier, do not directly provide amino acids and vitamins, they would allow organisms to synthesize essential metabolites at lower metabolic cost and could lead to precursor auxotrophy (the ‘black queen' hypothesis (Morris *et al.*, 2012)). Further analysis of marine genomes may improve our ability to link metabolite production to the demands of different types of auxotrophic organisms but clearly exchange of metabolites, as proposed here, would be mutually beneficial to microbes in a high-density consortium.

## Conclusions

**Figure 5:**
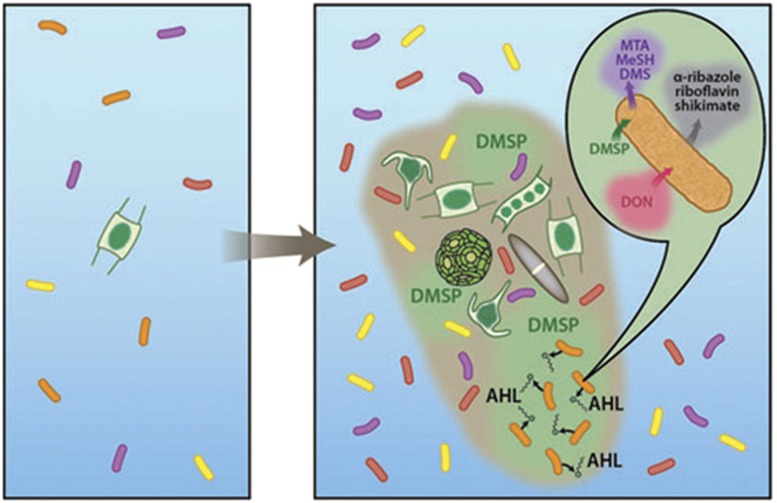
Visual interpretation of *R. pomeroyi*'s response to DMSP. The left-hand panel portrays the diffuse oligotrophic ocean populated by free-living organisms. In contrast, the right-hand panel shows dying plankton cells coalescing to form a colloid or particle composed of, and exuding, organic matter. The phytoplankton cells leak DMSP, which triggers production of a signaling molecule in *R. pomeroyi* (orange cells). Once the AHL is present at a sufficient concentration, an array of metabolic shifts occur, including reduced intracellular glutamine which may cause increased uptake of dissolved organic nitrogen (DON), increased output of high-value metabolites such as alpha-ribazole, riboflavin, and shikimate, and release of sulfur metabolites such as methanethiol (MeSH; Supplementary Figure S1c), dimethylsulfide (DMS; Supplementary Figure S1c), and MTA (image credit: Jack Cook, WHOI).

Differences in the phylogenetic diversity of free-living versus surface-associated microbial populations have been well-documented (DeLong *et al.*, 1993). However, many bacteria may switch between these two lifestyles, and the factors governing this switch are only beginning to be understood (Grossart, 2010). Our work expands on these studies by showing that specific metabolites, such as algal-derived DMSP, can trigger metabolic shifts that could be expressed under high population densities (Figure 5). If groups of bacteria are using some phytoplankton-derived metabolites as signals (Seyedsayamdost *et al.*, 2011), rates of particle degradation, and more broadly of carbon remineralization, may be intimately tied to particle composition and source. These results emphasize the need to understand the molecular-level composition of organic matter in the ocean because a single molecule, such as DMSP, can alter the metabolism of organisms that encounter it, with likely impact on their biogeochemical function.
