# Supplementary material for: Evidence for quorum sensing and differential metabolite production by a marine bacterium in response to DMSP
Source: ISME J. 2016 Feb 16;10(9):2304–16. doi: 10.1038/ismej.2016.6 (PMC4989321; doi:10.1038/ismej.2016.6)
Supplement: Supplementary Information [file ismej20166x1.docx]

Supplementary Information

**SI Methods**

**General details.** All chemicals were purchased from Sigma-Aldrich unless otherwise indicated. DMSP was purchased from Research Plus, Inc. Methanethiol, ammonium chloride, sodium phosphate, ferrous chloride, ammonium hydroxide, hydrochloric acid (trace metal grade), acetonitrile (optima grade), and methanol (optima grade) were purchased from Fisher Scientific. D3-glutamic acid was purchased from Cambridge Isotopes, d4-4-hydroxybenzoic acid from CDN Isotopes, and d5-sodium taurocholate from Toronto Research Chemicals through Fisher Scientific. We used water purified by a Milli-Q system (Millipore) for all solutions (resistivity 18.2 MΩ•cm @ 25 °C, TOC < 1 µM). All glassware was acid washed and combusted in an oven at 460˚C for at least five hours. All plasticware and media stock solutions were autoclaved before use. In the DMSP and propionate experiment the organic substrates were sterilized by filtering the stock solution through a 0.2 µm nylon syringe filter. *Ruegeria pomeroyi* DSS-3 [DSM 15171] was purchased from ATCC (Catalog #700808).

**Bacterial Cultures.** The defined medium was made with 0.2 μm-filtered seawater collected near high tide from Vineyard Sound (MA) in September 2013. The media was made with either 1 mM sodium propionate or 0.6 mM DMSP (3 mM carbon in both cases), 4 mM ammonium chloride, 30 nM monosodium phosphate, 100 nM ferrous chloride-ethylenediaminetetraacetic acid, 100 nM zinc chloride, 100 nM manganese(II) chloride, 1 nM cobalt(II) chloride, and 1 mL / L medium of f/2 vitamin solution. The bacteria were grown in 250 mL Erlenmeyer flasks (120 mL media) with glass wool in the neck in the dark at 23˚C. The experimental samples were inoculated with exponentially-growing *R. pomeroyi* that had been pre-conditioned in the target media for two transfers. At each time point two duplicate flasks were sacrificed for both treatments. Media blanks for each substrate were extracted at 0, 40, and 72 hours. Cultures were sacrificed at 0, 32, 38(propionate)/43(DMSP), 48, 60, and 72 hours. The third time point was offset between the propionate and DMSP treatments so that the same cell abundance was sampled in each treatment. These time points captured exponential and stationary growth phases in both experiments. Each culture was filtered through a 0.2 µm filter (Omnipore, EMD Millipore) using a glass vacuum filtration rig.

The cell concentration was monitored at 6-10 hour intervals throughout the experiment. Optical density was measured using a Thermo Electron Corporation Nicolet Evolution 300 Spectrophotometer set to 660 nm. This was calibrated to cells/mL using DAPI stained cell counts from cultures grown on the same media to make a five-point calibration curve.

**Intracellular Metabolite Extraction** (adapted from Rabinowitz and Kimball, 2007). Filters were placed in cryovials and frozen at -80˚C immediately after filtration. Subsequently, three quarters of each filter was weighed and cut into small pieces with methanol rinsed scissors on combusted aluminum foil. The pieces of the filter were placed in an 8 mL combusted amber glass vial and 1 mL of -20˚C 40:40:20 acetonitrile:methanol:water + 0.1 M formic acid was added to each vial. 25 µL of 1 µg/mL deuterated standard mix (d3-glutamic acid, d4-4-hydroxybenzoic acid, and d5-taurocholate) was added as extraction recovery standards. The vials were vortexed to shake apart filter pieces and fully expose them to the ice-cold solvent. They were sonicated for 10 minutes and the extract was transferred to micro-centrifuge tubes using Pasteur pipettes. The filter pieces were rinsed with 200 µL of extraction solvent and the rinse was added to the micro-centrifuge tubes as well. The extracts were centrifuged at 20,000 × *g* for 5 minutes. The supernatant was transferred to new 8 mL amber glass vials leaving behind any scraps of filter or cellular detritus. The extracts were neutralized with 26 μL of 6 M ammonium hydroxide and dried down in a vacufuge. The samples were reconstituted in 495 μL 95:5 water:acetonitrile and 5 μL of 5 μg/mL deuterated biotin (injection standard). The solution (100 µL) was placed in a glass insert in an autosampler vial for targeted metabolomic analysis.

From this same solution 250 μL was removed and placed in a new vial, dried down, and brought up to 500 μL with 0.01 M HCl in water. The solution was pulled through a 50 mg Agilent Bond Elut PPL cartridge that was pre-rinsed with 1 mL methanol. The cartridge was rinsed with 2 mL 0.01 M aqueous HCl, dried for five minutes by pulling air through the cartridge, and then the sample was eluted from the cartridge with 1 mL methanol. The samples were dried down in a vacufuge and reconstituted in 250 µL of 95:5 water:acetonitrile for untargeted analysis.

The percentage of the filter-extracted carbon retained once it had been re-extracted with a PPL cartridge was 0.1-1.9%. These values reflect the bulk carbon extracted and not the extraction efficiency of individual molecules, which may have higher extraction efficiencies depending on their molecular characteristics.

**Extracellular Metabolite Extraction.** The media filtrate was acidified to pH 2-3 with 12 M HCl (Dittmar *et al.*, 2008; after modification by Longnecker, 2015). It was then pulled through a methanol-rinsed 50 mg Agilent Bond Elut PPL (styrene divinyl benzene polymer) cartridge. The cartridge was rinsed with 2 mL 0.01 M HCl, and the metabolites were eluted with 1 mL methanol (adapted from Dittmar *et al.*, 2008). It was dried down in a vacufuge and reconstituted to a total volume of 1 mL as the targeted intracellular samples were above. The percentage of carbon extracted from the media using solid phase extraction was 2.6-18.8%. The low percentages may be related to the fact that most of the dissolved organic carbon in the media was either propionate or DMSP, neither of which are retained well on PPL cartridges (W. Johnson, unpublished results).

**Mass Spectrometry.** All samples were pre-separated with the same liquid chromatography method on a Phenomenex C18 reversed phase column (Synergi Fusion, 2.1 x 150 mm, 4 µm). Eluents were (A) Milli-Q water with 0.1% formic acid and (B) acetonitrile with 0.1% formic acid. The gradient included: hold at 5% B for 2 minutes; ramp to 65% B for 16 minutes; ramp to 100% B for 7 minutes and hold for 8 min. The column was re-equilibrated with the starting ratio of eluents for 8.5 min in between analyses.

Untargeted analyses were conducted using a hybrid linear ion trap - 7T Fourier transform ion cyclotron resonance mass spectrometer (FT-ICR-MS; LTQ FT Ultra, Thermo Scientific) with an electrospray ionization source. The instrument was externally calibrated weekly with a standard mix from the manufacturer. In parallel to the high resolution full MS scan in the FT-ICR cell (R = 100,000 defined at 400 *m/z*), the top four most abundant ions that exceeded 500 counts in the ion trap were fragmented. For the intracellular analysis, between every six samples the following sequence was run: Milli-Q blank, a quality control standard mix, Milli-Q blank, two pooled samples (one for conditioning and one for feature validation).

The targeted method used a Thermo Scientific triple quadrupole mass spectrometer with a heated electrospray ionization source. A mixture containing the standards for all of the targeted metabolites was run at 0.5, 1, 5, 10, 50, 100, 250, and 500 ng/mL concentrations to provide an external calibration curve for relative quantification. For the extracellular samples a 500 ng/mL standard mix was run approximately every six samples as quality control with a Milli-Q blank run before and after. In the intracellular samples a pooled sample of all the experimental samples was run after the second Milli-Q sample to re-condition the column.

**Data Processing.** The targeted data was processed using Xcalibur (Thermo Scientific software). Standard curves were assessed individually and low quality peaks removed. The media controls were subtracted from the intracellular samples and used as a starting point reference for the extracellular samples.

All untargeted analysis files were converted from Thermo RAW files to mzML files using MSConvert (Chambers *et al.*, 2012). The resulting data was processed using XCMS (Smith *et al.*, 2006; Tautenhahn *et al.*, 2008; Benton *et al.*, 2010) and CAMERA (Kuhl *et al.*, 2012). Peak picking and alignment were carried out separately for the intracellular and extracellular samples. In negative ion mode the peak threshold was set to an absolute intensity of 1000 in MSConvert before processing in XCMS. XCMS settings included: method = centWave, ppm = 2 (negative ion mode), 3 (positive ion mode), signal-to-noise = 10. The retention time correction setting and group.density were also used. Fragmentation mass spectra had a signal-to-noise threshold of 1. For the intracellular samples, pooled samples were used as an additional quality control. The average of each feature in the pooled samples or the experimental samples had to be greater than 1000. The coefficient of variance (100*standard deviation of feature/mean of feature) for each feature in the experimental samples and the pooled samples was calculated and only features that had a higher coefficient of variance in the experimental samples than in the pooled samples were retained for consideration (Vinaixa *et al.*, 2012).

The list of features defined by a retention time and *m/z* was further refined by removing any features found in the samples whose average was less than five times higher than the average of all the media blanks. In addition, features had to be present in both biological replicates at two different sampling points. This accounted for compounds that might be found in the seawater but were also being produced by the bacteria. To compare overall differences in metabolite composition in the samples, non-metric multidimensional scaling based on Bray-Curtis distance matrices was used.

Features in the untargeted data, consisting of a unique *m/z* and retention time, were putatively identified by matching the *m/z* to metabolites in the METLIN database (Smith *et al.*, 2005) or using MetFrag (Wolf *et al.*, 2010) to match the *m/z* to metabolites listed in KEGG (Kanehisa *et al.*, 2014). If a fragmentation spectrum was available for a feature, this spectrum was compared to experimentally-generated fragmentation patterns in the METLIN database or to *in silico* derived fragmentation patterns from MetFrag.

**DOC & TOC Measurements.** 10 mL of whole media was diluted with Milli-Q water to 40 mL and acidified to pH 2-3 for total organic carbon (TOC) analysis. 10 mL of 0.2-μm filtered media was diluted to 40 mL with MilliQ water and acidified to pH 2-3 for dissolved organic carbon (DOC) analysis. Samples were stored at 4˚C until analysis. The non-purgeable organic carbon was measured using a Shimadzu TOC-V_CSH_ Total Organic Carbon Analyzer. A five-point calibration curve made with potassium hydrogen phthalate was used and blanks were run regularly. Comparisons to standards from D. Hansell (University of Miami) were made daily.

**Gas Chromatography-PFPD**. 2 mL of the unfiltered media was sealed in a serum vial. A 200-μL sample of headspace was taken with a syringe and injected into a gas chromatograph with an Alltech AT-Sulfur capillary 0.32 mm ID column and an Ol Corp. pulsed flame photometric detector (adapted from Levine *et al.*, 2012). The presence of methanethiol and dimethylsulfide was confirmed with commercial standards.

Benton HP, Want EJ, Ebbels TMD. (2010). Correction of mass calibration gaps in liquid chromatography-mass spectrometry metabolomics data. *Bioinformatics* **26**:2488–2489.

Chambers MC, Maclean B, Burke R, Amodei D, Ruderman DL, Neumann S, *et al.* (2012). A cross-platform toolkit for mass spectrometry and proteomics. *Nat Biotechnol* **30**:918–920.

Dittmar T, Koch B, Hertkorn N, Kattner G. (2008). A simple and efficient method for the solid-phase extraction of dissolved organic matter (SPE-DOM) from seawater. *Limnol Oceanogr Methods* **6**:230–235.

González JM, Covert JS, Whitman WB, Henriksen JR, Mayer F, Scharf B, *et al.* (2003). *Silicibacter pomeroyi* sp. nov. and *Roseovarius nubinhibens* sp. nov., dimethylsulfoniopropionate-demethylating bacteria from marine environments. *Int J Syst Evol Microbiol* **53**:1261–1269.

Kanehisa M, Goto S, Sato Y, Kawashima M, Furumichi M, Tanabe M. (2014). Data, information, knowledge and principle: back to metabolism in KEGG. *Nucleic Acids Res* **42**:199–205.

Kuhl C, Tautenhahn R, Bottcher C, Larson TR, Neumann S. (2012). CAMERA: An integrated strategy for compound spectra extraction and annotation of liquid chromatography/mass spectrometry data sets. *Anal Chem* **84**:283–289.

Levine NM, Varaljay VA, Toole DA, Dacey JWH, Doney SC, Moran MA. (2012). Environmental, biochemical and genetic drivers of DMSP degradation and DMS production in the Sargasso Sea. *Environ Microbiol* **14**:1210–1223.

Longnecker K. (2015). Dissolved organic matter in newly formed sea ice and surface seawater. *Geochim Cosmochim Acta* **171**:39–49.

O’Toole GA, Trzebiatowski JR, Escalante-Semerena JC. (1994). The cobC gene of *Salmonella typhimurium* Codes for a novel phosphatase involved in the assembly of the nucleotide loop of cobalamin. *J Biol Chem* **269**:26503–26511.

Rabinowitz JD, Kimball E. (2007). Acidic acetonitrile for cellular metabolome extraction from *Escherichia coli*. *Anal Chem* **79**:6167–6173.

Reisch CR, Crabb WM, Gifford SM, Teng Q, Stoudemayer MJ, Moran MA, *et al.* (2013). Metabolism of dimethylsulphoniopropionate by *Ruegeria pomeroyi* DSS-3. *Mol Microbiol* **89**:774–791.

Reisch CR, Moran MA, Whitman WB. (2011). Bacterial catabolism of dimethylsulfoniopropionate (DMSP). *Front Microbiol* **2**:172–184.

Smith CA, Maille GO, Want EJ, Qin C, Trauger SA, Brandon TR, *et al.* (2005). A metabolite mass spectral database. *Ther Drug Monit* **27**:747–751.

Smith CA, Want EJ, O’Maille G, Abagyan R, Siuzdak G. (2006). XCMS: processing mass spectrometry data for metabolite profiling using nonlinear peak alignment, matching, and identification. *Anal Chem* **78**:779–787.

Sumner LW, Amberg A, Barrett D, Beale MH, Beger R, Daykin CA, *et al.* (2007). Proposed minimum reporting standards for chemical analysis: Chemical Analysis Working Group (CAWG) Metabolomics Standards Initiative (MSI). *Metabolomics* **3**:211–221.

Tautenhahn R, Böttcher C, Neumann S. (2008). Highly sensitive feature detection for high resolution LC/MS. *BMC Bioinformatics* **9**:504–520.

Vinaixa M, Samino S, Saez I, Duran J, Guinovart JJ, Yanes O. (2012). A guideline to univariate statistical analysis for LC/MS-based untargeted metabolomics-derived data. *Metabolites* **2**:775–795.

Wolf S, Schmidt S, Müller-Hannemann M, Neumann S. (2010). In silico fragmentation for computer assisted identification of metabolite mass spectra. *BMC Bioinformatics* **11**:148–160.

Table S1. Total number of features based on *m/z* and retention time in the untargeted data. Blank-corrected refers to the number of features remaining once the controls were accounted for as described in the supplementary methods. The final column lists the number of blank corrected features that had fragmentation patterns associated with them.

| **Ion Mode** |  | **Total** | **Blank Corrected** | **Has a Fragmentation Pattern** |
| --- | --- | --- | --- | --- |
| Positive | Intracellular | 9220 | 1117 | 307 |
|  | Extracellular | 11724 | 2379 | 448 |
| Negative | Intracellular | 4646 | 897 | 229 |
|  | Extracellular | 13543 | 1686 | 396 |

**Table S2: Characteristics of intra- and extracellular metabolites observed in two substrate treatments.** Columns 2, 3, 4: the number of features found in both substrates compared to the features found in one treatment. Columns 5, 6, 7: the percentage of features at 60 hours in the DMSP treatment whose concentration (in peak area / cell) was 5 times higher than in the propionate treatment, features 5 times higher in the propionate treatment, feature peak area was approximately the same in both treatments (equal), Columns 8, 9, 10: the same data as columns 5-7, at 72 hours.

| **Ion Mode** | | **Presence/absence** | | | **Concentrations (Peak area / cell)** | | | | | |
| --- | --- | --- | --- | --- | --- | --- | --- | --- | --- | --- |
| Intracellular | | | | | | | | | | |
|  | DMSP | | Overlap | Propionate | 60 hours | | | 72 hours | | |
|  |  |  |  |  | DMSP 5x | Propionate 5x | Equal | DMSP 5x | Propionate 5x | Equal |
| Neg | 4% | | 43% | 53% | 9% | 86% | 5% | 6% | 86% | 8% |
| Pos | 1% | | 96% | 3% | 17% | 64% | 19% | 13% | 66% | 21% |
| Extracellular | | | | | | | | | | |
|  | DMSP | | Overlap | Propionate | 60 hours | | | 72 hours | | |
|  |  |  |  |  | DMSP 5x | Propionate 5x | Equal | DMSP 5x | Propionate 5x | Equal |
| Neg | 1% | | 98% | 1% | 19% | 28% | 53% | 24% | 43% | 33% |
| Pos | 6% | | 89% | 5% | 23% | 36% | 41% | 22% | 51% | 27% |

**Table S3. Matches of *m/z* and fragments for putatively identified compounds discussed in the paper.** Column 2: the exact mass adjusted for either negative ion mode ([M-H]^-^) or positive ion mode ([M+H]^+^), Column 3: measured *m/z* of the feature in the untargeted data, Column 4: the ppm difference between the feature *m/z* and the adjusted exact mass, Column 5: the measured retention time (RT) of the feature in minutes, Column 6: possible fragments calculated from MetFrag that matched those of the feature, Column 7: fragments and their intensity relative to the highest peak (after the comma) from the METLIN database that were generated in an Q-TOF mass spectrometer, Column 8: Measured fragments of the feature observed in the experiment and their intensity relative to the highest peak (after the comma), Column 9: The confidence ranking of identification based on the reporting standards proposed by Sumner et al. (2007) where 1 is the highest confidence identification.

| **Compound** | **Exact mass** | **Feature *m/z*** | **ppm match** | **RT** | **Predicted MetFrag fragments** | **METLIN** | **Measured fragments** | **Confidence Ranking** |
| --- | --- | --- | --- | --- | --- | --- | --- | --- |
| 3-dehydro shikimate | 171.0299 | 171.0300 | 0.6 | 4.8 | 127.0  83.0  141.0 | 171.0, 100  127.0, 26  109, 7  81.0, 1 | 127.0, 100  83.0, 21  102.9, 9  141.0, 3  171.0, 50 | 2 |
| shikimate | 173.0455 | 173.0457 | 1.2 | 7.7 | 129.0  127.0  111.0 | 173.0, 100  93.0, 9  111.0, 7  137.0, 4  129.0, 0 | 129.0, 100  127.0, 39  111.0, 15  173.0, 70 | 2 |
| chorismate | 225.0405 | 225.0406 | 0.4 | 8.3 | -- | -- | -- | 3 |
| phenylpyruvate | 163.0401 | 163.0402 | 0.6 | 10.2 | -- | -- | -- | 3 |
| 3-(methylthio)propionate | 119.0172 | 119.0173 | 0.8 | 7.8 int/  7.2 ext | -- | -- | -- | 3 |
| *N*-(3-oxotetradecanoyl)-L-homoserine lactone | 326.2326 | 326.2327 | 0.3 | 23.7 | -- | 326.2, 100  102.1, 97  225.2, 76 | 327.0, 7  101.9, 24  225.1, 100  270.1, 9 | 1 |
| α-ribazole* | 279.1339 | 279.1340 | 0.4 | 6.6 | 147.0 | -- | 147.0, 100 | 2 |

*Fragmentation pattern from literature also supports this identification (O’Toole *et al.*, 1994).

**Table S4. Initial intermediates produced by DMSP degradation.** There are two pathways for DMSP degradation: demethylation/demethiolation and cleavage (Reisch *et al.*, 2011, 2013). R. pomeroyi has been demonstrated to utilize both pathways (González *et al.*, 2003). The demethylation/demethiolation pathway produces methanethiol which can be lost as a gas or incorporated into biomass. The cleavage pathway cleaves dimethylsulfide first before utilizing the remainder of the molecule. Both pathways ultimately lead to central carbon metabolism. The table summarizes the intermediates detected in each pathway. “Detected?” column indicates whether the metabolite was detected in any of our methods.

| DMSP (Demethylation/Demethiolation)   | | DMSP (Cleavage)   | |
| --- | --- | --- | --- |
| Intermediate | Detected? | Intermediate | Detected? |
| 3-(methylthio)propionate | Yes (no MS2) | acrylate | No (too small) |
| 5-methyl-THF | No | Dimethylsulfide (DMS) | Yes |
| MMPA-CoA | No | acryloyl-CoA | No |
| MTA-CoA | No |  |  |
| acetaldehyde | No * |  |  |
| Methanethiol (MeSH) | Yes |  |  |

*I Acetaldehyde will not ionize under our ESI conditions and it is too small to be detected by the FT-ICR MS with our parameters.

**SI Figure Captions:**

**Fig. S1. *R. pomeroyi* growth, carbon remineralization, and organosulfur gas production. a,** Average growth curves of *R. pomeroyi* growing on DMSP (red) and propionate (blue). The black diamonds indicate the time points where sampling occurred. The error bars are the standard deviation for the two biological replicates. It is unclear why there is an offset in cell density between the two treatments but is perhaps due to different metabolic efficiencies for catabolism of the two carbon substrates. **b,** Total organic carbon (TOC; dark red, DMSP treatment and dark blue, propionate treatment) and dissolved organic carbon (DOC; orange, DMSP treatment and light blue, propionate treatment) concentrations. Cell-free media controls are in faded colors (red-orange, DMSP and blue propionate) and show no decline in organic carbon during the experiment. Error bars represent one standard deviation for the biological replicates. The target concentration for both carbon substrates was 3 mM but some loss of the substrates may have occurred with filter sterilization. **c,** Abundance of dimethylsulfide (DMS) and methanethiol (MeSH) in the headspace of the initial DMSP cultures and after 72 hours. The presence of both gases confirms that both the demethylation pathway and the lyase pathway (see Table S4) were used by *R. pomeroyi* for DMSP catabolism. In **S1b** the DMSP treatment has less organic carbon at the outset than propionate and that difference increases by the final timepoint at 72 hours. Subtracting the initial difference still leaves a difference in both TOC and DOC of 0.3 mM at 72 hours. This difference is due to the loss of DMS and MeSH as shown in **S1c** that only occurs in the DMSP treatment. If we calculate the total loss of DOC over the course of the experiment for each substrate, the DMSP treatment lost 1.4 mM carbon and the propionate treatment lost 1.1 mM carbon. However, to compare bacterial utilization, it may be more relevant to consider the moles of each molecule respired rather than the total carbon because the portion of DMSP that enters primary carbon metabolism is the same 3-carbon unit as propionate. DMS and MeSH are lost from the medium as gases. We can approximate this by normalizing to the number of carbon atoms in each molecule (5 for DMSP and 3 for propionate). We calculate a loss of 0.3 mM for DMSP and 0.4 mM for propionate. Thus the draw-down of moles of each organic substrate is not very different, especially assuming that there would be some difference in efficiency between the pathways.

**Fig. S2 A heatmap of all the targeted intracellular metabolite concentrations in the two treatments.** Averages of biological replicates are compared at each time point (0 hours-72 hours). (Red): concentrations are higher in the DMSP treatment increasing from dark to light; (Blue) concentrations were higher in the propionate treatment increasing from dark/greenish to cyan; (Black) the concentrations in the two substrate treatments were similar; (Gray) metabolites were not detected in either treatment. Metabolites with a significant difference in concentration between the substrate treatments are indicated with (*) and the *p*-value from an ANOVA test is reported.

**Fig. S3. MTA concentrations in the 90% propionate / 10% DMSP experiment and the relationship between MTA response in the targeted and untargeted methods. a,** Extracellular concentrations of MTA during stationary growth phase, normalized to cell abundance. *R. pomeroyi* was grown on media with 100% propionate, 100% DMSP, and 10% DMSP / 90% propionate. The total carbon added to each treatment was the same (3 mM). **b,** Comparison of extracellular MTA measured in the targeted method versus the untargeted method. The linear trend indicates that relative MTA response was similar on both instruments. There was a significant correlation between the two methods (Pearson correlation coefficient, r = 0.9778, p << 0.0001). Red is the DMSP treatment, blue is the propionate treatment.

**Fig. S4. Non-metric multidimensional scaling plots for extracellular samples based on a Bray-Curtis distance matrix of peak area normalized to filtrate volume.** For both positive and negative ion modes, the dominant factor is time although there is some clustering based on substrate. The relative percent of variation explained by each axis is included in the label. Each marker represents a sample and more similar samples appear closer to one another in this plot. Squares represent propionate samples and triangles represent DMSP samples.

**Fig. S5: Overview of MTA production and degradation.** MTA is a by-product of two metabolic pathways. One produces an acyl homoserine lactone (left) and the other converts putrescine to spermidine (right). MTA and an AHL were measured extracellularly at higher concentrations in the DMSP treatment. The *R. pomeroyi* genome lacks this pathway for spermidine/spermine synthesis that produces MTA and instead contains the genes for an alternate pathway requiring the enzymes carboxyspermidine dehydrogenase and carboxyspermidine decarboxylase to convert putrescine to spermidine. Only the first gene (S-methyl-5´-thioadenosine phosphorylase) of the classic methionine salvage pathway has been annotated in the *R. pomeroyi* genome.

**Fig. S6: End-products of the biosynthetic pathways that require chorismate and chorismate biosynthesis.** Tyrosine was not included in the targeted method and was not detected in the untargeted method. Phenylalanine, tryptophan, 4-aminobenzoic acid, and 2,3-dihydroxybenzoic acid were all quantified in the targeted method. If present, the cell-normalized concentrations were similar between both treatments. Red: DMSP treatment, Blue: propionate treatment, Black: compound was not detected in either treatment.
