# Supplementary figures and images for: Evidence for quorum sensing and differential metabolite production by a marine bacterium in response to DMSP
Source: ISME J. 2016 Feb 16;10(9):2304–16. doi: 10.1038/ismej.2016.6 (PMC4989321; doi:10.1038/ismej.2016.6)

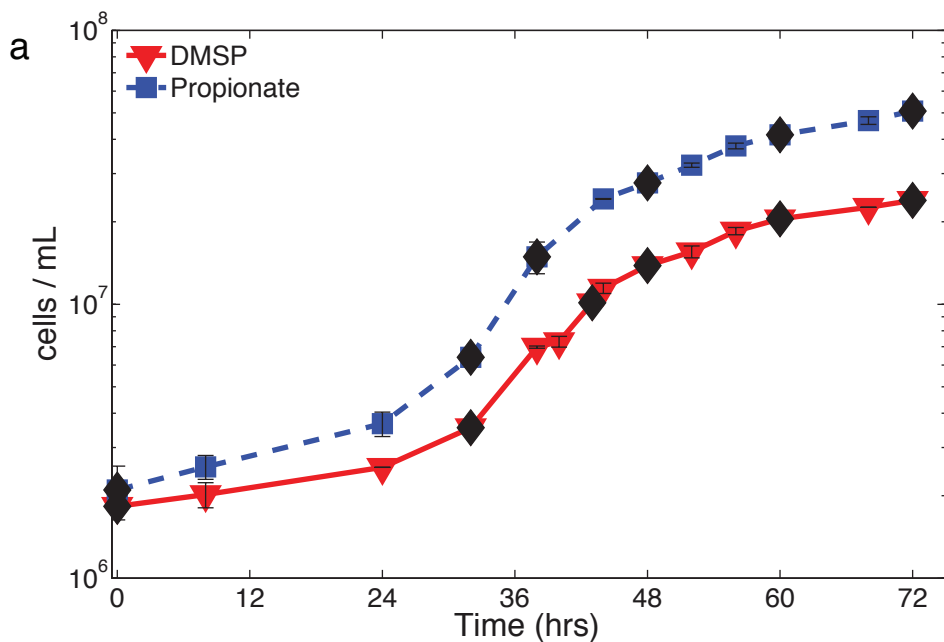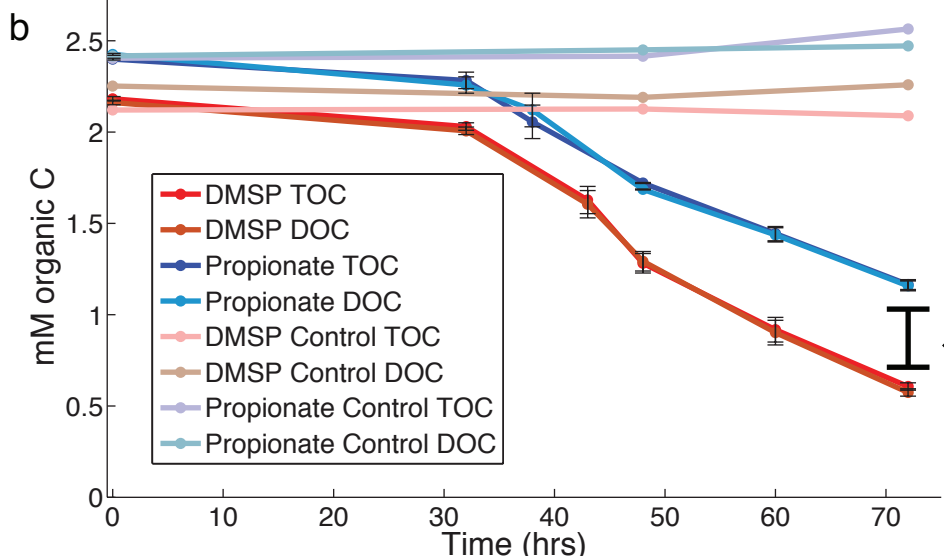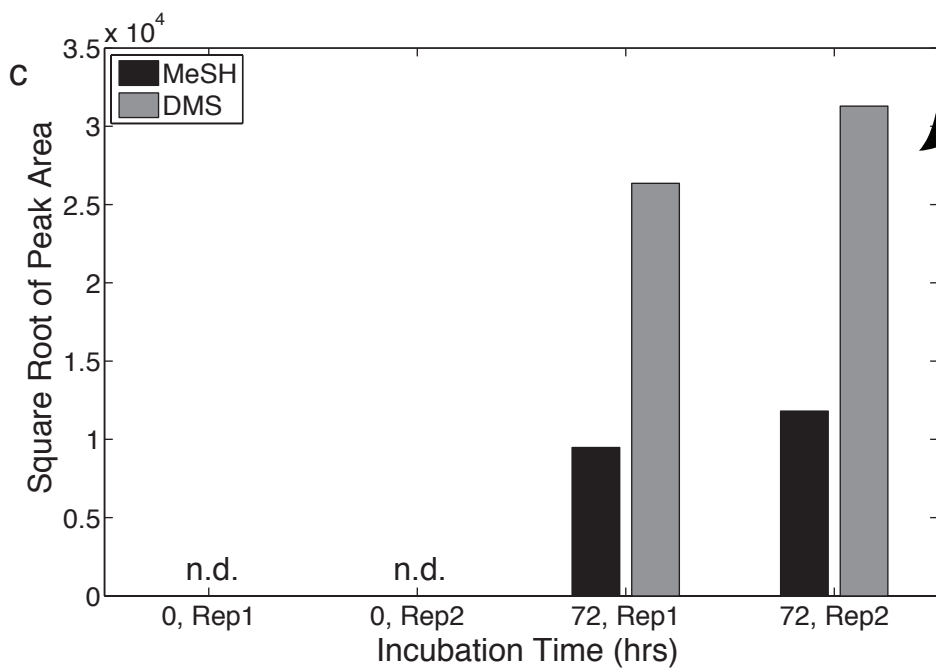

Supplement: Supplementary Figure S1 [file ismej20166x2.pdf]

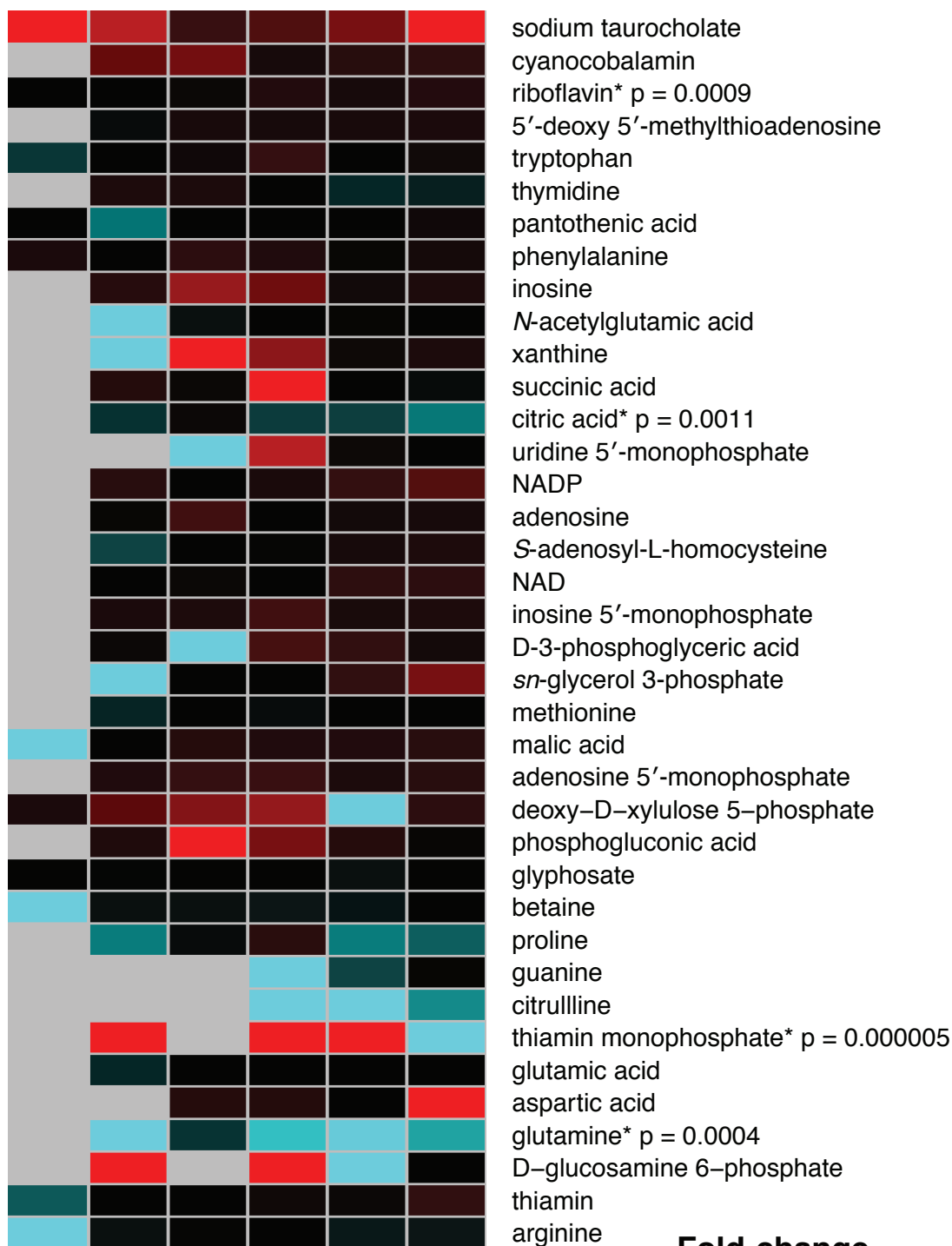

**Fold-change**

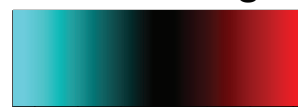

64 16 4 0 4 16 64

up in propionate up in DMSP

Supplement: Supplementary Figure S2 [file ismej20166x3.pdf]

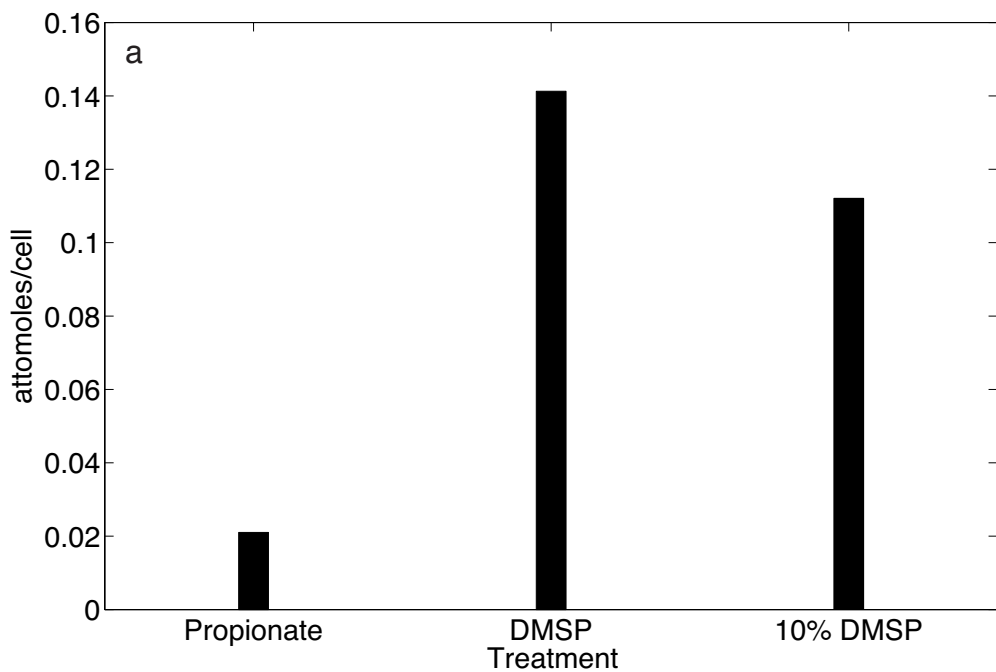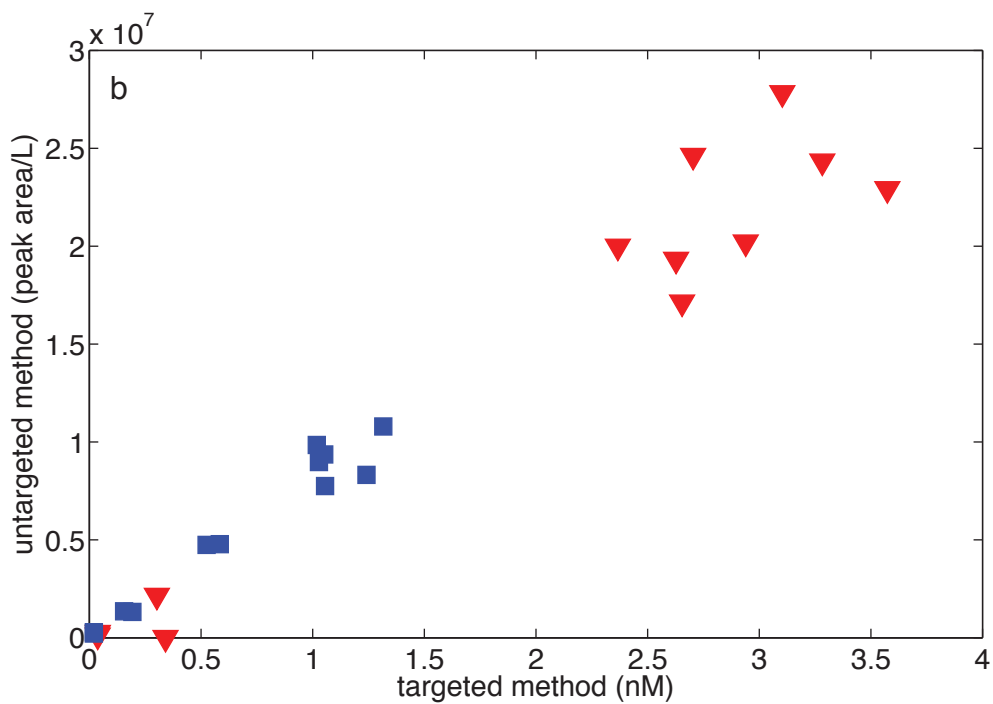

Supplement: Supplementary Figure S3 [file ismej20166x4.pdf]

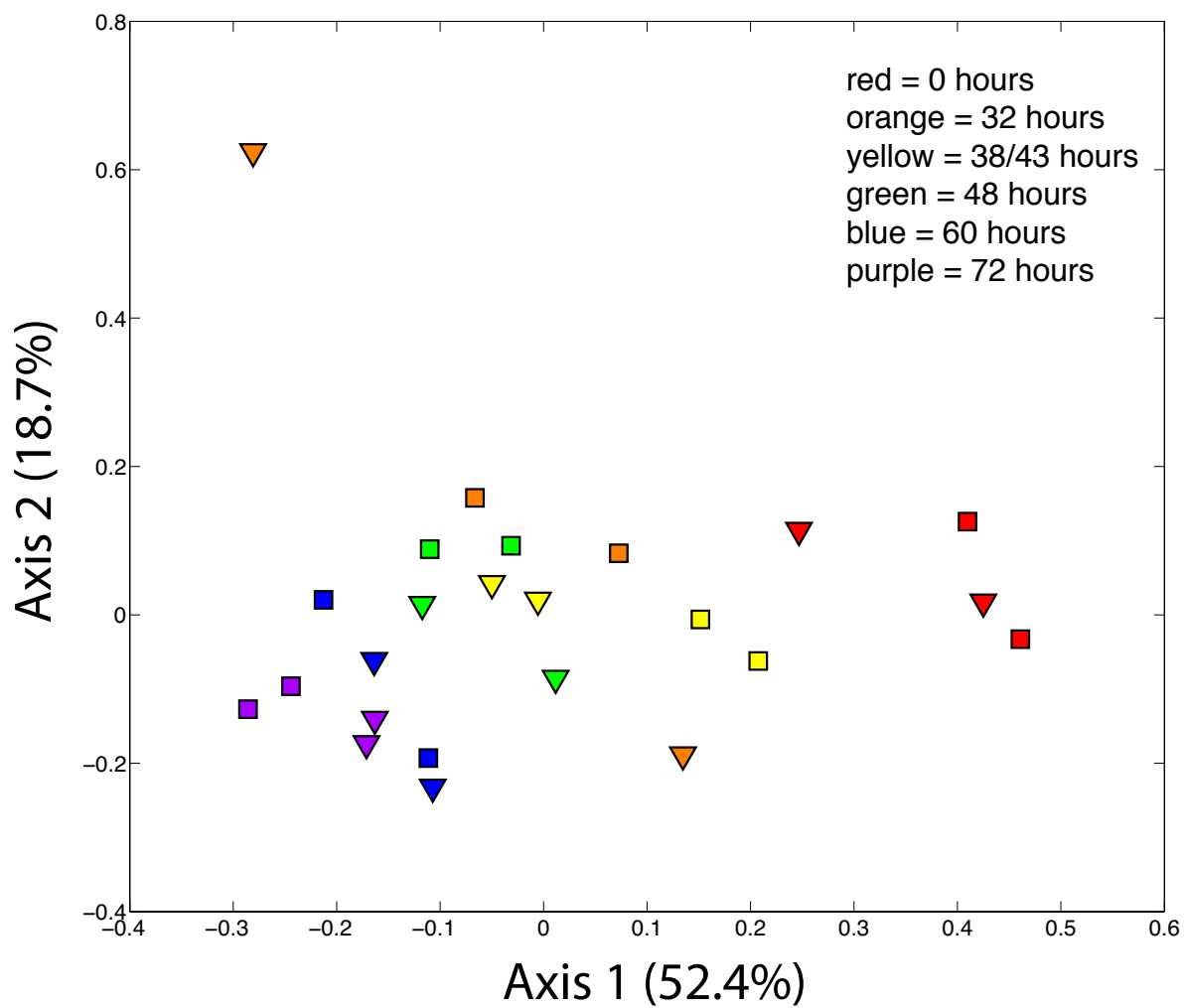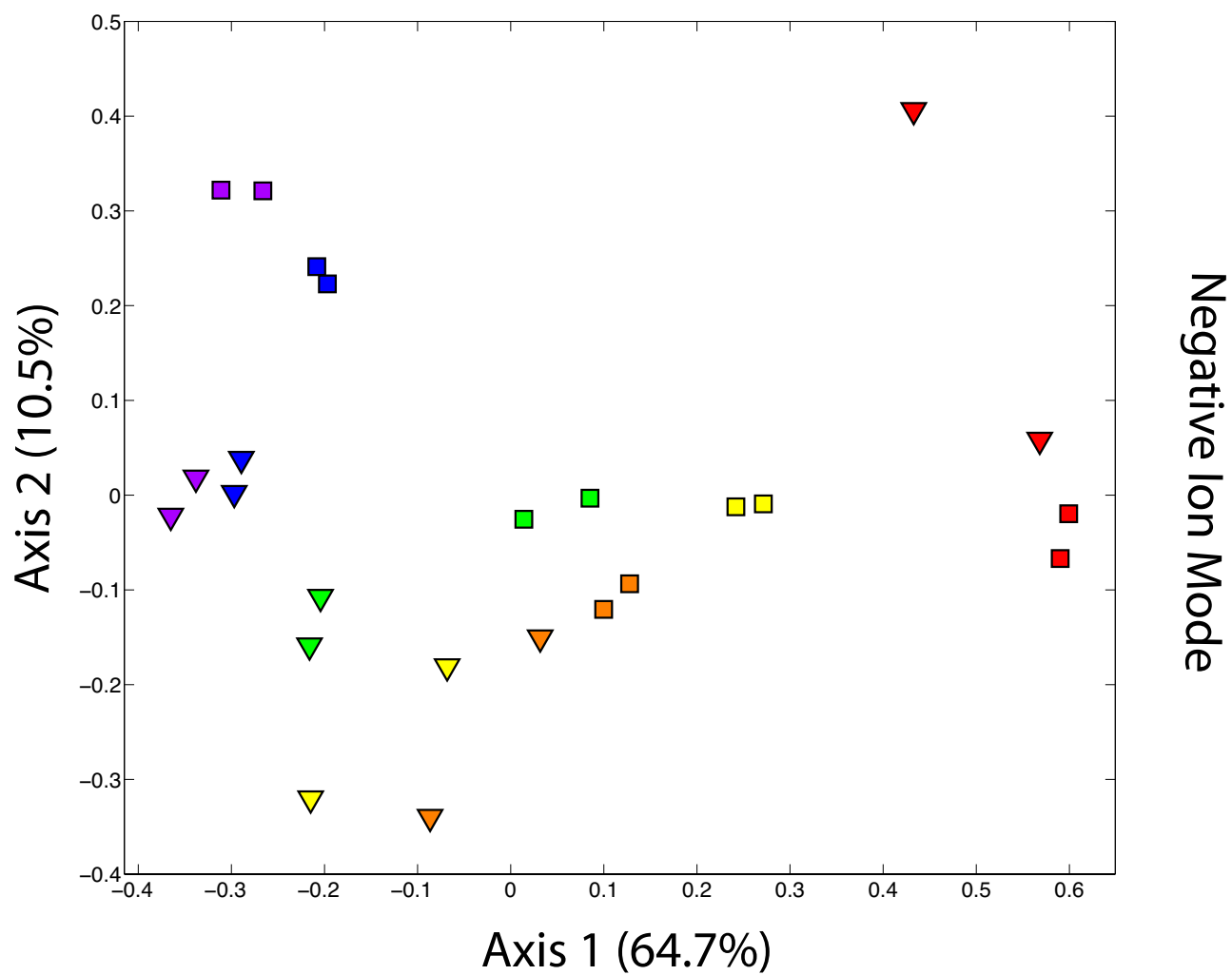

Supplement: Supplementary Figure S4 [file ismej20166x5.pdf]

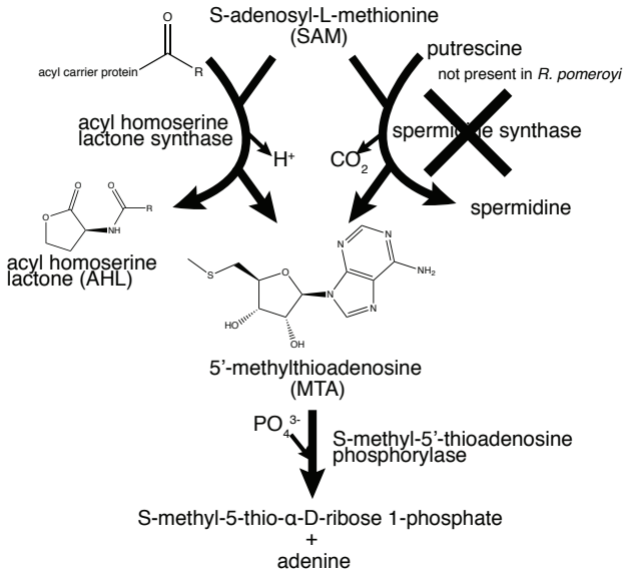

Supplement: Supplementary Figure S5 [file ismej20166x6.pdf]
