# Supplementary material for: Evidence for quorum sensing and differential metabolite production by a marine bacterium in response to DMSP
Source: ISME J. 2016 Feb 16;10(9):2304–16. doi: 10.1038/ismej.2016.6 (PMC4989321; doi:10.1038/ismej.2016.6)
Supplement: Supplementary Figure S6 [file ismej20166x7.pdf]

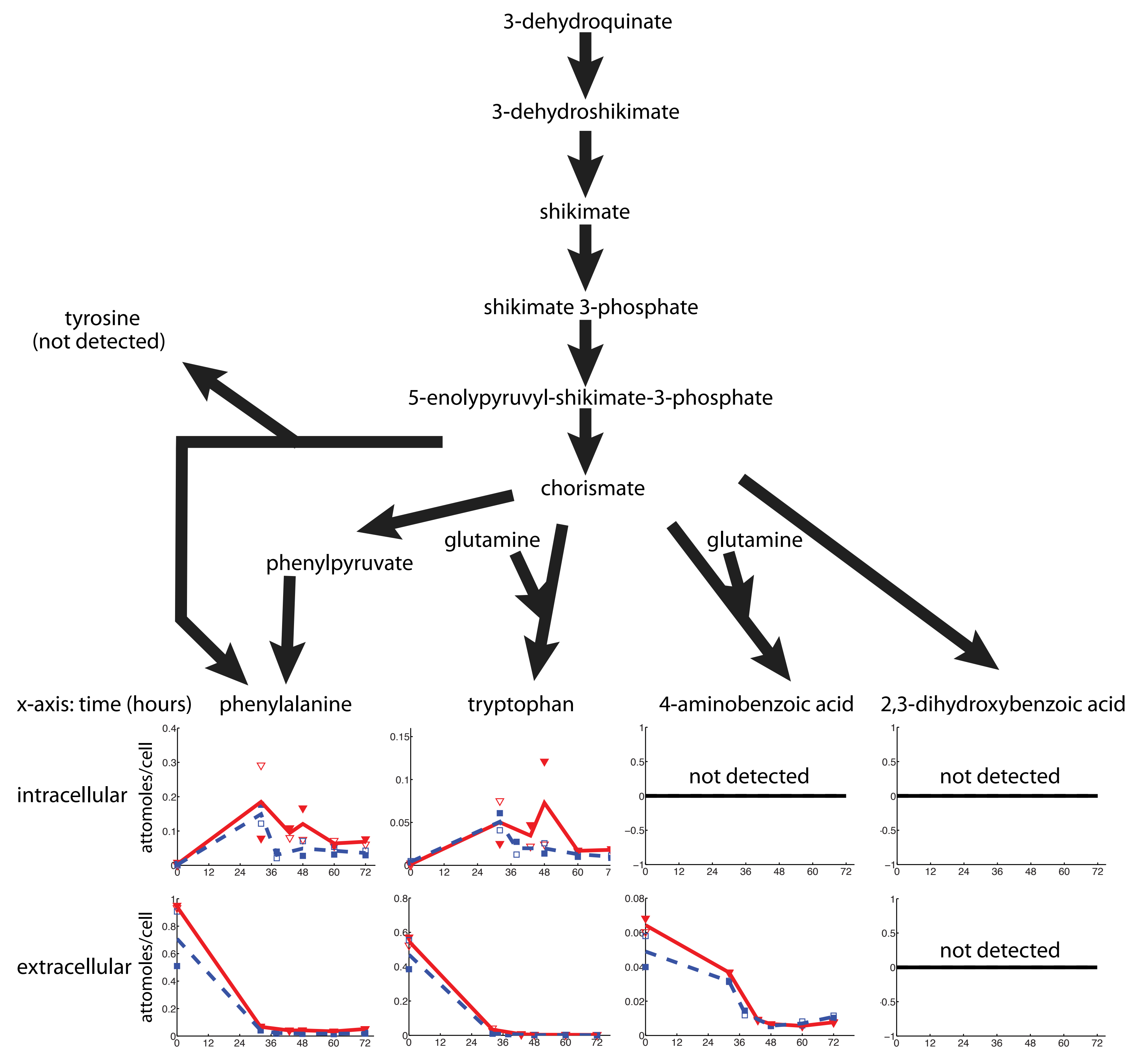
3-dehydroquinate
3-dehydroshikimate
shikimate
shikimate 3-phosphate
5-enolypyruvyl-shikimate-3-phosphate
tyrosine
(not detected)
chorismate
glutamine
phenylpyruvate
glutamine
x-axis: time (hours)
phenylalanine
tryptophan
4-aminobenzoic acid
2,3-dihydroxybenzoic acid
intracellular
extracellular
attomoles/cell
attomoles/cell
not detected
not detected
not detected
